# *Candida auris* Containment Responses in Health Care Facilities that Provide Hemodialysis Services — New Jersey, North Carolina, South Carolina, and Tennessee, 2020–2023

**DOI:** 10.15585/mmwr.mm7425a1

**Published:** 2025-07-10

**Authors:** Alexandra Kurutz, Gabriel K. Innes, Adrienne Sherman, Lakisha Kelley, Kendalyn Stephens, Patricia Kopp, Benjamin Cohen, Erin Haynes, Christopher Wilson, Simone Godwin

**Affiliations:** ^1^Tennessee Department of Health; ^2^New Jersey Department of Health; ^3^North Carolina Department of Health and Human Services; ^4^South Carolina Department of Public Health.

**Summary**

**What is already known about this topic?**

*Candida auris*, a frequently multidrug-resistant fungal pathogen, can spread within health care facilities. Dialysis facilities face particular infection prevention and control (IPC) challenges because their patients require complex medical care and frequent invasive procedures.

**What is added by this report?**

In five facilities providing dialysis in four states, six patients infected or colonized with *C. auris* received dialysis for up to 4 months without transmission to other patients. Five of the facilities had no knowledge of the patients’ *C. auris* status and had implemented standard dialysis IPC only.

**What are the implications for public health practice?**

Adherence to standard dialysis IPC practices appeared sufficient to prevent transmission of *C. auris* among dialysis patients. More evidence is needed to understand the prevalence of and risk factors associated with *C. auris* transmission in the dialysis setting.

## Abstract

*Candida auris*, a frequently multidrug-resistant fungal pathogen, poses an urgent public health threat due to its potential to spread within and between health care facilities. Facilities that offer dialysis services might face particular challenges in preventing and containing *C. auris* and other multidrug-resistant pathogens, given the frequent use of invasive treatments in an immune-compromised patient population. During 2020–2023, in five separate facilities providing dialysis care across four states (New Jersey, North Carolina, South Carolina, and Tennessee), six patients infected or colonized with *C. auris* received dialysis treatment for up to 4 months; five patients’ *C. auris* status was unknown to the facilities treating them. A review of public health response efforts carried out in these facilities was conducted. Before the facilities became aware of these patients’ *C. auris* status, they implemented recommended standard but not *C. auris*–specific infection prevention and control (IPC) measures for the dialysis setting. Colonization testing of 174 potentially exposed patient contacts identified one additional patient whose previously detected *C. auris* colonization was not known to the dialysis facility, but no additional positive test results. Lapses in communication among health care facilities (e.g., acute care, long-term care, and dialysis) and public health jurisdictions posed a significant impediment to containment response efforts by most participating states. Adherence to standard dialysis IPC practices appeared to enable safe provision of dialysis to patients with *C. auris* colonization or infection without transmission to other dialysis patients. However, improved interfacility communication regarding patients’ infection or colonization status with multidrug-resistant organisms is needed to ensure prompt implementation of all recommended IPC practices. More evidence is needed to understand the prevalence of and risk factors associated with *C. auris* transmission in the dialysis setting.

## Introduction

*Candida auris* is an emerging fungal pathogen that poses an urgent public health threat because it is frequently resistant to multiple drugs and has the ability to spread quickly within health care facilities (*1*). Because of the underlying morbidity and immunosuppression of patients who are susceptible to clinical infection, and the limited treatment options, an estimated 39% of *C. auris* infections are fatal (*2*). Patients with end-stage kidney disease are at risk for *C. auris* colonization or infection because they often require highly complex inpatient care, use invasive medical devices, have immune-compromising medical conditions, and regularly receive broad-spectrum antimicrobial drugs. The transmissibility and high levels of antifungal resistance that are characteristic of *C. auris* set it apart from most other *Candida *species (*3*).

Patients colonized with *C. auris* often harbor the pathogen indefinitely without ever experiencing symptoms; therefore, timely identification of colonization, ensuring effective cleaning of the equipment and the environment using an approved environmental disinfectant, such as Environmental Protection Agency List P products,* and application of appropriate transmission-based precautions are crucial to containing *C. auris* and minimizing the number and extent of outbreaks (*4*). No studies have examined *C. auris* transmission in the dialysis setting. To highlight challenges and considerations in the care of persons infected or colonized with *C. auris* in the dialysis setting, a review of *C. auris* containment responses was conducted after identification of patients with *C. auris* who received dialysis at five facilities in four states during 2020–2023, some without the facilities' prior awareness of their *C. auris* status.

## Methods

### Data Source and Study Design

CDC facilitates quarterly telephone calls with health departments to answer infection control questions and establish best practices for management of *C. auris* given the difficulty of managing the infection. During those calls in 2023–2024, health departments in four states (New Jersey, North Carolina, South Carolina, and Tennessee) reported that persons who received a positive *C. auris* laboratory test result had received on-site dialysis services at five facilities, some without the facilities’ prior awareness of their *C. auris* status. Containment-driven responses were conducted by health departments at the five facilities where these persons had received on-site dialysis services *C. auris* investigation and containment responses involving at least one round of colonization testing of potentially exposed patients were self-reported by state health departments. Responses described in this report were led by state health departments in the four states during 2020–2023 (*5*). This activity was conducted under respective New Jersey, North Carolina, South Carolina, and Tennessee public health authority as a surveillance activity necessary for public health work and therefore did not require institutional review board review.

A colonization case was defined as detection of *C. auris* through polymerase chain reaction testing or culture testing of axilla, groin, or nares swab specimens collected as part of facility surveillance activities from a patient receiving dialysis within a facility. A clinical case was defined as detection of *C. auris* in a specimen from any other sterile or nonsterile site obtained as part of clinical care from a patient receiving dialysis in a facility; identification of clinical cases was based on specimen source and not on the patient’s clinical signs and symptoms. The first reported cases in a facility were designated the index cases.^†^^,^^§^

### Response to the Index Cases

After identification of an index case (colonization or clinical), state public health authorities facilitated the testing of other patients for *C. auris* colonization. An investigation was conducted to identify additional health care facilities where the index patient received care before, during, or after collection of the positive specimen. Colonization testing was recommended for potential contacts (i.e., persons who received dialysis in the same facility as the index patient on the same or following shift or who received inpatient care at the same facility or floor where the index patient received care). After detection of the index case, a colonization testing event was conducted by the facility at the earliest feasible date. In South Carolina and Tennessee, a follow-up colonization testing round was recommended 2 weeks after the first date, focusing on the same patient population.

### Specimen Collection and Testing

Colonization testing specimens were collected by swabbing the axilla, groin, or nares; procedures and specimen collection sites varied by state. All specimens for colonization testing were collected using flocked Eswabs (a liquid-based collection and transport system) and transferred in Amies transport mediums to the states’ respective Antimicrobial Resistance Laboratory Network regional laboratory, which conducted polymerase chain reaction testing. Colonization testing strategies varied based on state protocol, epidemiologic data, and type of health care setting. Patients not receiving dialysis were included in colonization testing in New Jersey and North Carolina. Because patients are considered to be indefinitely colonized after initial identification of *C. auris* colonization or clinical infection, index patients were not included in colonization testing.

## Results

### Facility Characteristics

During 2020–2023, four states initiated response activities after detection of six index patients who received dialysis, including three with *C. auris* colonization cases and three with clinical cases (Table 1). Facilities identified in the public health response (with response year) included one co-located acute care hospital or skilled nursing facility with inpatient and outpatient dialysis in South Carolina (2020), one skilled nursing facility with on-site inpatient and outpatient dialysis in New Jersey (2021), one outpatient dialysis facility in Tennessee (2023), one outpatient dialysis facility in North Carolina (2023), and one acute care hospital with inpatient dialysis in North Carolina (2023).

**TABLE 1:** Characteristics of containment responses for *Candida auris* in five dialysis facilities, by state and year ― New Jersey, North Carolina, South Carolina, and Tennessee, 2020–2023

Characteristic	State (yr)				
	South Carolina (2020)	New Jersey (2021)	Tennessee (2023)	North Carolina (2023)	
Facility setting	Co-located acute care hospital and skilled nursing facility; onsite inpatient and outpatient dialysis	Skilled nursing facility; onsite inpatient and outpatient dialysis	Outpatient dialysis	Outpatient dialysis	Acute care hospital; onsite inpatient dialysis
No. of index patients	1	1	1	2	1
Case type of index patients	Colonization	Colonization	Colonization	Clinical	Clinical
No. of colonization testing rounds conducted	2	1	2	1	1
No. of patients who received *C. auris* colonization testing	20	76*	26	35	17*
Specimen collection sites	Axilla and groin	Axilla, groin, and nares	Axilla and groin	Axilla	Axilla
Total no. (%) of positive colonization test results	0 (—)	0 (—)	1 (3.8)^†^	0 (—)	0 (—)
No. of mos from first positive test result in an index patient to implementation of transmission-based precautions	<1	1	4	<1	1
Reported barriers to containment	Lack of out-of-state interfacility communication	Delayed facility communication with state health department because no point of contact for infection prevention and control; high staff member turnover; and lack of interfacility communication	Lack of in-state interfacility communication	Lack of privacy during colonization testing; lack of interfacility communication from previous state	NA
Colonization testing population	Patients on same dialysis shift as index patient	Facilitywide (all residents)	Patients on same outpatient dialysis shift and subsequent shift as index patient	Patients on same shift, treatment pod, or dialysis station or with same attending staff member as index patient ≤1 mo of index specimen collection date	Patients admitted to same acute care hospital floor as index patient or treated at inpatient dialysis center ≤1 mo of index specimen collection date

**Abbreviation:** NA = not applicable.

* Patients surveyed included those who were and were not receiving dialysis.

^†^ Patient had previously been identified with *C. auris* colonization, but the dialysis facility was not aware of this.

### Characteristics of Index Patients

Among the six index patients, the mean age was 64 years (range = 38–79 years), three patients had *C. auris* colonization at the time of the response, and three had clinical cases (one each who received a positive test result from a blood, urine, or wound specimen). Two index patients were identified at a single North Carolina outpatient dialysis facility; these patients had both already received health care in states other than North Carolina and had both received care at the same North Carolina acute care/critical access hospital (ACH) but at different times.

### Response Activities

In each state, identification of the index case triggered a containment response in accordance with CDC’s Interim Guidance for a Public Health Response to Contain Novel or Targeted Multidrug-resistant Organisms.^¶^ Containment responses involved 1) notifying the health care facilities where the index patients had health care exposure; 2) providing guidance on infection prevention and control (IPC), including recommending implementation of transmission-based precautions and proper cleaning and disinfection practices; 3) conducting colonization testing of health care contacts; and 4) recommending an on-site infection control assessment.

### Colonization Testing

State health departments used scenario-specific recommendations for testing, and adherence to state-issued containment recommendations varied by facility. Each of the four states conducted at least one colonization testing event, with a combined total of 174 potential contacts. Colonization testing in the New Jersey skilled nursing facility and the North Carolina ACH included a combination of dialysis and nondialysis patients. Colonization testing identified no new colonization cases. One Tennessee patient who received testing as part of the containment response received a positive colonization test result. Investigation revealed that this patient had already received positive *C. auris* colonization test results 4 months earlier; however, the patient’s results were not reported to the dialysis facility at admission.

### Facility Containment Activities

Before dialysis-specific *C. auris* IPC measures were published by the CDC, early recommendations to dialysis facilities treating patients colonized or infected with *C. auris* were generalized from other health care settings. These recommendations included changing personal protective equipment (PPE) (including gown and gloves) between patient encounters, thoroughly cleaning and disinfecting the dialysis station between patient treatments using disinfectant products from List P (or List K** if appropriate), scheduling the patients’ dialysis during the last shift of the day, when patient traffic is lower and directly before daily terminal cleaning, and providing dialysis for patients with *C. auris* adjacent to as few other dialysis stations as possible (e.g., at the end or corner of the unit) (Table 2). Public health recommendations did not require isolation rooms for dialysis of patients with *C. auris*.

**TABLE 2:** Standard and *Candida auris*–specific infection prevention and control recommendations for five dialysis facilities **—** New Jersey, North Carolina, South Carolina, and Tennessee, 2020–2023

Procedure	Recommendation
Standard dialysis IPC practices*	
General	Dispose of, use only for a single patient, or disinfect after use for items taken into the dialysis station (e.g., chairs, side tables, and machines); clearly designate clean areas for the preparation, handling, and storage of medications and unused supplies.
Hand hygiene	Wear disposable gloves when caring for patients or touching their equipment at the dialysis station; remove gloves and wash hands between each patient and station encounter.
Medication management	Only use medications taken to a patient’s station for that specific patient, and do not return unused medications to a common clean area; do not carry multiple-dose medication vials from station to station, and prepare the vials in a central clean area; do not puncture intravenous medication vials labeled for single use more than once, and do not pool residual medication from two or more vials into a single vial; do not use common medication carts to deliver medications to patients; and clearly designate clean areas for the preparation, handling, and storage of medications and unused supplies.
Patient equipment management	Use external venous and arterial pressure transducer filters and protectors for each patient treatment, and change them between each patient treatment; clean and disinfect dialysis station between patient treatments (using hospital disinfectants registered by the Environmental Protection Agency); discard all fluid, and clean and disinfect all surfaces and containers associated with the prime waste; cap dialyzer ports and clamp tubing for dialyzers and blood tubing that will be reprocessed; and place used dialyzers and tubing in leakproof containers for transport from station to reprocessing or disposal area.
Additional *Candida auris*–specific dialysis IPC measures*	
Education	Inform and educate personnel about the presence of a patient with *C. auris* and the need for specific IPC measures.
Personal protective equipment use	Wear gowns and gloves using proper donning and doffing techniques when caring for patients with *C. auris* or touching items at the dialysis station; remove gowns and gloves, dispose of them carefully, and perform hand hygiene when leaving the patient's station.
Minimize exposure of other patients	Provide dialysis for colonized or infected patients at a station with as few adjacent stations as possible (e.g., at the end or corner of the unit), and consider dialyzing the patient on the last shift of the day.
Management of reusable equipment	Thoroughly clean and disinfect the dialysis station between patients using products approved for use against *C. auris*; properly clean and disinfect reusable equipment brought to the dialysis station after each use.
Patient transfers	If the patient is transferred to another health care facility, inform the receiving facility of the patient's *C. auris* status.

**Abbreviation**: IPC = infection prevention and control.

* This is not a complete list of standard dialysis IPC practices or *C. auris*–specific IPC procedures. Complete guidance is available at CDC | Dialysis Safety. Guidelines, Recommendations and Resources and CDC | *Candida auris *Infection Control Guidance.

### Challenges with Treating Patients with *C. auris* and Implementing Containment Response

In four states, the index patients had received dialysis treatment for ≤4 months since their first test-positive collection date, in some instances without any additional control measures other than standard dialysis IPC practices.^††^ Gaps in interfacility communication regarding transferred patients’ *C. auris* status occurred in three states, including, in one instance, across state lines.

Outside the context of these described containment responses, dialysis facilities expressed concern to state health departments about their ability to safely treat patients with *C. auris*, citing the presence of communal treatment areas, limited availability of isolation rooms, and the vulnerability of their patient populations. Two states reported that patients with *C. auris* had been declined treatment at other dialysis facilities based on these concerns. One state completed assessments of two facilities’ IPC practices using CDC’s Infection Control Assessment and Response tool,^§§^ and no gaps were identified for either facility.

Adherence to state-issued containment recommendations varied by facility. Facilities in two states that were engaged in response activities reported delayed implementation of colonization testing. In these responses, lack of designated points of contact and high staff member turnover rates were cited as barriers to arranging and conducting colonization testing. In one case, the coordinating health department referred the facility to the state’s licensing body, citing IPC deficiencies. One state reported that colonization testing to identify colonized or infected patients was well-received by the facility’s clinical teams.

## Discussion

In addition to the standard precautions followed by nondialysis facilities (e.g., inpatient acute care facilities), recommendations guide dialysis facilities to follow additional precautions (referred to as standard dialysis IPC practices) because of the increased risk for contamination with blood and pathogenic microorganisms in these facilities. Examples of these additional precautions include frequent cleaning of equipment and the environment with bleach solution (observed by one state that performed on-site IPC assessments), restriction of shared common supplies and instruments, and prohibiting use of a shared medication cart.

Minimizing exposure of dialysis patients with *C. auris* colonization or infection to other patients through strategic scheduling and spacing, disinfection with products on the Environmental Protection Agency’s List P (or List K if appropriate), and ensuring appropriate use of PPE (including always changing gowns and gloves when transitioning between patients) could reduce transmission of *C. auris* in dialysis settings (*1*). Current public health recommendations do not require isolation rooms for dialysis of patients with *C. auris*; therefore, unavailability of isolation rooms should not impede the delivery of dialysis services to this patient population.

In five instances across four states, patients with *C. auris* colonization or clinical infection received dialysis treatment for ≤4 months, in some instances without facility knowledge of the patients’ *C. auris* status or application of additional *C. auris*–specific dialysis facility precautions beyond standard dialysis IPC practices. Even when the patients’ status was not communicated by upstream facilities, resulting in longer duration of receiving dialysis treatments without additional precautions, transmission within the facility was not observed. The exact timing of index patient colonization could not be confidently determined in these instances; therefore, it cannot be ruled out that their original exposure to *C. auris* occurred within a dialysis facility. In North Carolina, a 2-week overlap occurred in dialysis treatment between two index patients identified at the same facility; however, the patient with the earlier positive culture date was being treated with *C. auris* precautions in place during the entire overlap period. In addition, these patients did have the same *C. auris* clade but were deemed not related through the number of single nucleotide polymorphisms.

*C. auris* transmission was not detected in these responses despite the application of only standard dialysis IPC practices. The absence of observed transmission in the assessed dialysis facilities might be explained by their strict adherence to standard IPC guidance for that setting; however, the sample size was small, and the generalizability of findings was limited. Still, the identification of patients requiring additional dialysis-specific *C. auris* IPC measures,^¶¶^ including those with asymptomatic colonization, remains an important component of prevention of transmission of multidrug-resistant organisms in health care settings.

### Limitations

The findings in this report are subject to at least four limitations. First, a limited number of states and facilities were involved in the study, and dialysis settings varied, with not all involved facilities being representative of the settings where most chronic, ambulatory hemodialysis is performed, limiting the generalizability of these findings (*6*,*7*). Second, the study was completed as a secondary analysis, which limited the type and extent of data collected. This fact is especially relevant to the paucity of data collected on the exact types of infection prevention precautions taken by the various dialysis facilities. Third, different specimen collection sites and colonization testing approaches were used for each state’s containment-driven responses, which could affect the reliability of test results because of lack of standardization. Finally, two of the participating facilities included a combination of dialysis and nondialysis patients in the colonization testing, and information was not available on the number of dialysis versus nondialysis patients included in the colonization surveys at these facilities.

### Implications for Public Health Practice

Coordination of case management among dialysis facilities and transferring facilities could improve interfacility communication regarding patients’ infection or colonization with multidrug-resistant organisms and help to ensure prompt implementation of all recommended IPC practices. This study suggests that with adherence to appropriate precautions, dialysis can be safely provided to patients regardless of their *C. auris* status. Further studies are needed to better understand the prevalence and risk factors associated with *C. auris* transmission in the dialysis setting.

## Footnotes

*
United States Environmental Protection Agency. EPA’s Registered Antimicrobial Products Effective Against *Candida auris* [List P]

†
CDC | National Notifiable Diseases Surveillance System (NNDSS). *Candida auris*. 2023 Case Definition

§ The index patients described in this report received testing for *C*. *auris* after dialysis had been ongoing. Patients receiving outpatient dialysis who are admitted to an acute care facility for any reason return to an outpatient facility after discharge from the acute care facility. Because of time constraints or incomplete interfacility communication, results of testing, such as that for *C. auris* colonization or infection might not be reported to the dialysis care team until a later date.

¶
CDC | Infection Control. Multidrug-resistant Organisms (MDRO) Management Guidelines

**
United States Environmental Protection Agency | EPA’s Registered Antimicrobial Products Effective Against *Clostridioides difficile* (*C. diff*) Spores [List K]

†† CDC | Dialysis Safety. Guidelines, Recommendations and Resources

§§ CDC | Healthcare–Associated Infections (HAIs). Infection Control Assessment and Response (ICAR) Tool for General Infection Prevention and Control (IPC) Across Settings

¶¶
CDC | *Candida auris *(*C. auris*). Infection Control Guidance: *Candida auris*
